# Effect of proper oral rehabilitation on general health of mandibulectomy patients

**DOI:** 10.1002/ccr3.373

**Published:** 2015-09-10

**Authors:** Ammar A Mustafa, Kais Raad, Nazih S Mustafa

**Affiliations:** 1 Faculty of Dentistry, The University of Hong KongHong Kong, China; 2 ISF Medical ClinicDoha, Qatar; 3 Faculty of Dentistry, International Islamic University MalaysiaKuantan, Malaysia

**Keywords:** Health-related quality of life, limited oral aperture, manidbulectomy, oral rehabilitation

## Abstract

Here, we aimed to assess whether postoperative oral rehabilitation for mandibulectomy patients is necessary to improve patients’ general health in terms of health-related quality of life.

## Introduction

Reduced oral aperture and mandibular opening are relatively common problems, which have a wide variety of causes 1. Limited access to the oral cavity can be problematic for patients and dental professionals 2. According to Glossary of Prosthodontics terms GPT 3, reduced oral aperture is defined as microstomia, which is a term used to describe any congenital or acquired reduction in the size of the oral opening that is severe enough to compromise esthetic, deglutition, nutrition, and quality of life 4.

The mandible is the key bone involved in face esthetic, mastication, and speech. Surgical resection of the mandible (known as a mandibulectomy) is often performed for tumors of the head and neck area, which should be followed by oral rehabilitation (i.e., occlusal adjustments and replacement of missing teeth and/or soft tissues, if involved) 5. The treatment of oral tumors such as squamous cell carcinomas may require mandibular resection to secure adequate margins 6. Segmental resection of the mandible leads to significant patient illness if not properly managed. Mandibulectomy can lead to loss of mandibular support to the teeth, inadequate mastication, impaired speech and disfigurement of the face 7.

### Statement of problem

According to the guidelines of the British Society for Disability and Oral Health, making an ordinary denture impression is extremely difficult or even impossible for patients with a limited mouth opening 8. In maxillo-facial surgical operations, especially when the mandible is involved, microstomia as a postoperative sequel is highly anticipated 9, which could affect the quality and quantity of dietary intake 10. Subsequently, the general health of the patient may deteriorate and negatively impact body weight and quality of life. The major Health-Related Quality of Life HRQL changes for head and neck cancer patients are observed within the first year after diagnosis. Dry mouth, altered senses, and impaired mastication are persistent, significant problems experienced by patients after treatment and can have serious influences on patients’ general health 11. There is a relationship between malnutrition (> or =;10% weight loss) and HRQL, and HRQL questionnaires and diagnoses are used to detect severe weight loss in patients with head and neck cancer 12.

Reduction in HRQL can be directly related to weight loss and malnutrition, with an improvement seen when dietary counseling and aggressive nutritional support is maintained during treatment 13. Dental professionals should be familiar with the differential diagnosis and the approaches for managing reduced oral aperture 14. Therefore, treatment should be planned carefully to overcome the difficulties that may interfere with the oral rehabilitation procedures and optimization of nutritional status should be considered a long-term priority 13. In this case study, we aimed to discuss the outcomes of oral rehabilitation for a patient with irregular reduced oral aperture as a result of mandibulectomy and to improve the patient's quality and quantity of dietary intake, thereby improving the patient's general health and quality of life.

## Case Report

**Figure 1:**
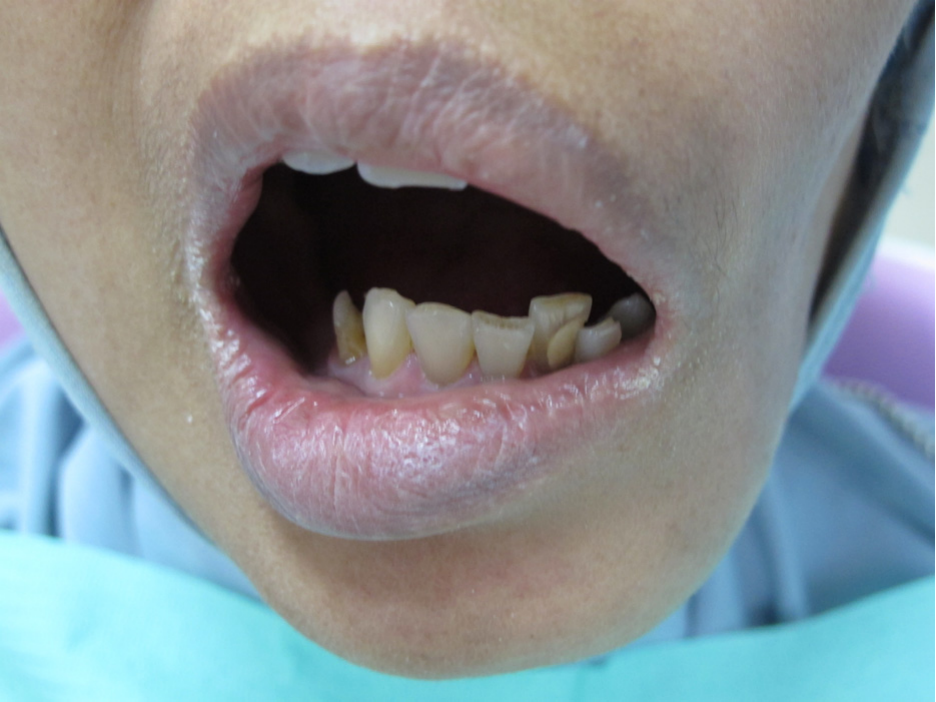
The patient was presented with irregular and limited mouth opening because of mandibulectomy.

**Figure 2:**
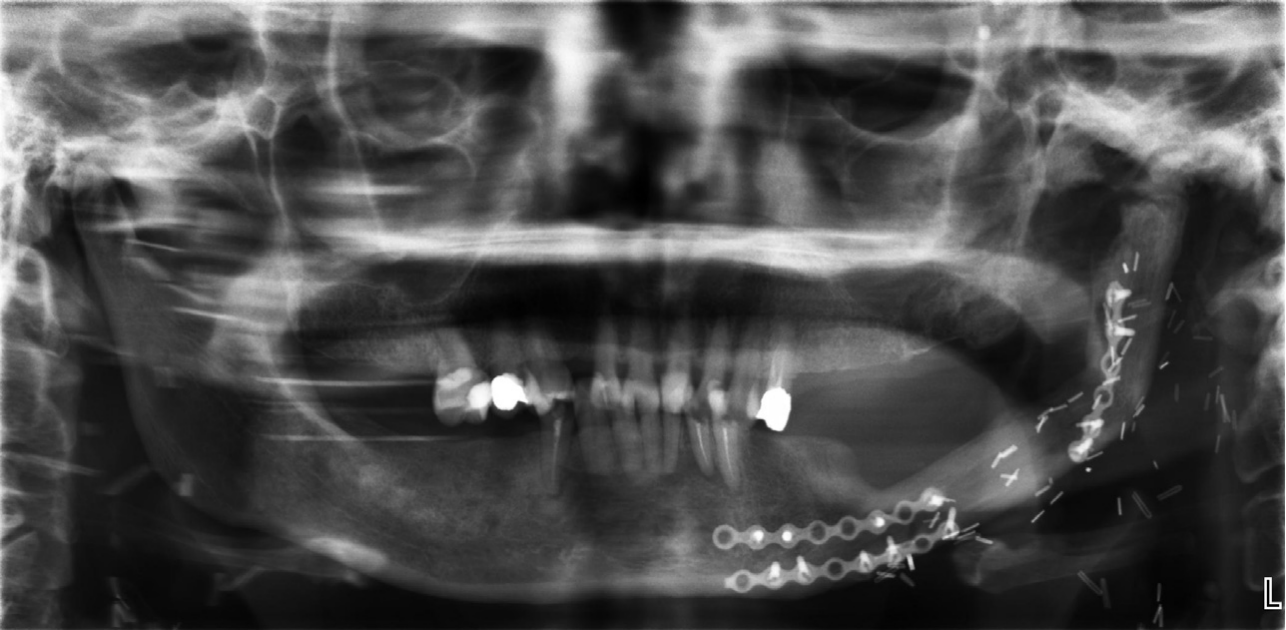
Orthopantomograph shows the marginal mandibulectomy for the left mandible with three plate fixers. *Note*: the pin-like radiopaque structures are actually golden pins used by the villagers as a traditional belief for healing.

**Figure 3:**
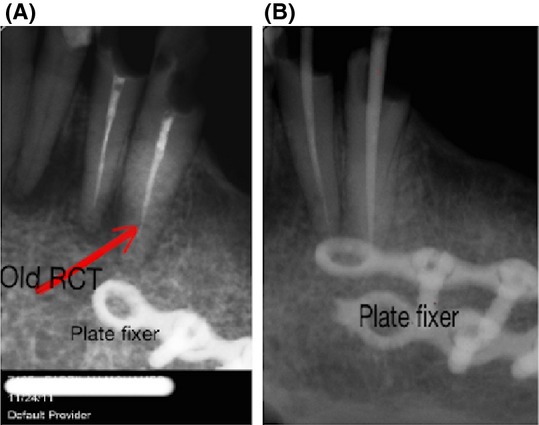
#34 and #35 were presented with a history of old deficient RCT. Note that the apexes are so close to the plate fixers. (A) Before oral rehabilitation, (B) During RCT as part of planned oral rehabilitation.

A 43-year-old Malay women suffering from reduced oral aperture due to recent mandiblectomy was referred to the prosthetic clinic at the Kulliyyah of Dentistry, IIUM-Malaysia. The average mouth opening was 35 mm for the diameter and 30 mm for the circumference 15. The principal complaint was fear of lesion recurrence due to rapid loss of weight after the mandibulectomy, with the patient under the false impression that the lesion was still active and affecting her general health. The patient was 40 kg in weight at the time of the first visit, which could be attributed to the continuous use of simple pureed food for a long period of time and an inability to chew properly. It was documented in the patient's medical records that no postoperative oral rehabilitations were performed. The mouth opening was not regular (i.e., the right side had a wider opening than the left side giving an irregular oblique mouth opening) (Fig.1). The patient presented with a history of squamous cell carcinoma of the left mandible. She had undergone surgical excision of the body of the mandible from the ramus to the area of the lower left premolars. The excised bone was replaced with a synthetic bone block. Orthopantomograph (OPG) and peri-apical X-rays revealed that the patient had lost the entire upper and lower molar teeth as a precaution prior to radiotherapy (Fig.2). However, the remaining premolar teeth were broken, with only the roots remaining. Peri-apical X-ray revealed that the apical parts of the remaining #33 and #34 teeth were so close to the metal plate fixers that extraction of these teeth was considered an extremely risky procedure (Fig.3). For the same reason and because of the limited remained bone, implant treatment was excluded. Therefore, the designated treatment was conventional upper and lower cobalt chromium removable partial dentures.

Based on history, clinical findings, radiographic examinations, and general health of the patient, a treatment plan was devised according to the following criteria: (1) the patient's motivation to control oral hygiene and to understand the importance of the current treatment; (2) pre-prosthetic oral preparation including scaling and root planing to the remaining teeth, restoration for the remaining natural teeth and root canal therapy for #33 and #34; (3) Construction of definitive cobalt-chromium removable partial over denture for the upper and lower jaws; and (4) nutritional screening and monitoring.

Root canal therapy was performed carefully as the apexes were very close to the bone-metal plate fixers (Fig.3). After assuring the accurate treatment of the canals, the retained roots were prepared as dome-shaped to be covered with metal coping for removable partial over denture. Nutritional screening was performed to identify the patient's malnourishment. Screening was conducted according to Roland & Paleri 8. Screening was repeated weekly. Weight was recorded at each visit at the department of Nutrition Science, Faculty of Allied Health Science IIUM-Malaysia.

The use of conventional impression stock trays was unmanageable because of the severely limited mouth opening. Therefore, heavy body putty silicone (Take 1 Hydrophilic vinyl putty material, Kerr, Germany) was used for preliminary impression making of the remaining teeth and the residual ridge. The primary impression was removed after setting, and checked for flawless registration of the anatomical landmarks. The putty/wash technique was performed using a very low viscosity siloxane (Extrude, Kerr). The preliminary impression was poured with type IV die stone.

**Figure 4:**
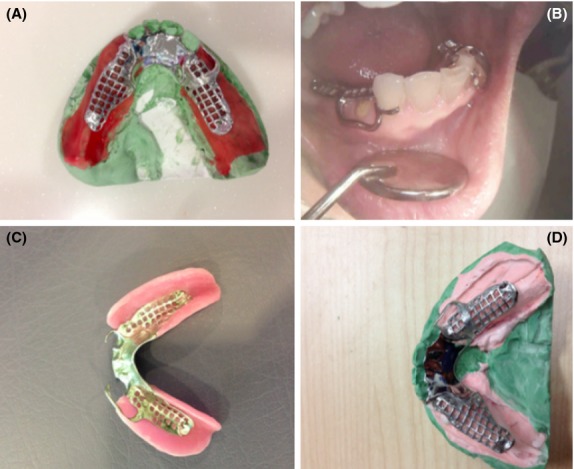
A) Metal framework on master cast, (B) Close fit tray-plast saddle to make altered cast technique, (C) and (D) Altered cast.

After registering the anatomical form of the residual ridge and the remaining teeth, functional form was achieved using an altered cast technique 16. The metal frameworks were tried to both the upper and lower jaws. Cold cure resin for custom-built impression trays (Tray Plast, Vertex, Netherlands) was used to construct close-fit saddles for the frameworks (Fig.4). After relining the saddles with tracing wax material, a functional impression was taken using medium body silicone (Extrude).

Because of the alteration in number and shape of the artificial teeth and in order to secure balanced contacts in eccentric positions beyond the range of the masticatory function, the maxillo-mandibular relations were performed according to a modified neutro-centric concept of occlusion. The neutro-centric concept was decided to minimize the tipping forces by reducing the effect of the incline plane contact between the maxillary and mandibular dentures. The occlusal surfaces of the premolar teeth were reshaped from flat to a cusp steep inclination of less than 33°. The occlusal surfaces of the molar teeth were adjusted to be flat.

The plane of occlusion was adjusted parallel to the denture foundation area. The molar teeth were set to a slightly flat plane of occlusion until the first molar because of the irregularity in the shape of the residual ridge after the surgery. The artificial teeth were reshaped and shortened occluso-cervically to accommodate the limited inter-ridge space and the occlusal surfaces were set to 0° on registering the maxillo-mandibular relations.

The patient was advised and trained to chop soft food for the first week, rather than chewing it, because of the semi-flat designed occlusal surfaces of the artificial teeth. Weeks later, the patient was trained to chew food normally and to begin eating hard food. Training involved giving the patient 1 cm^3^ carrot cubes and large pieces of peanuts 17,18.

**Figure 5:**
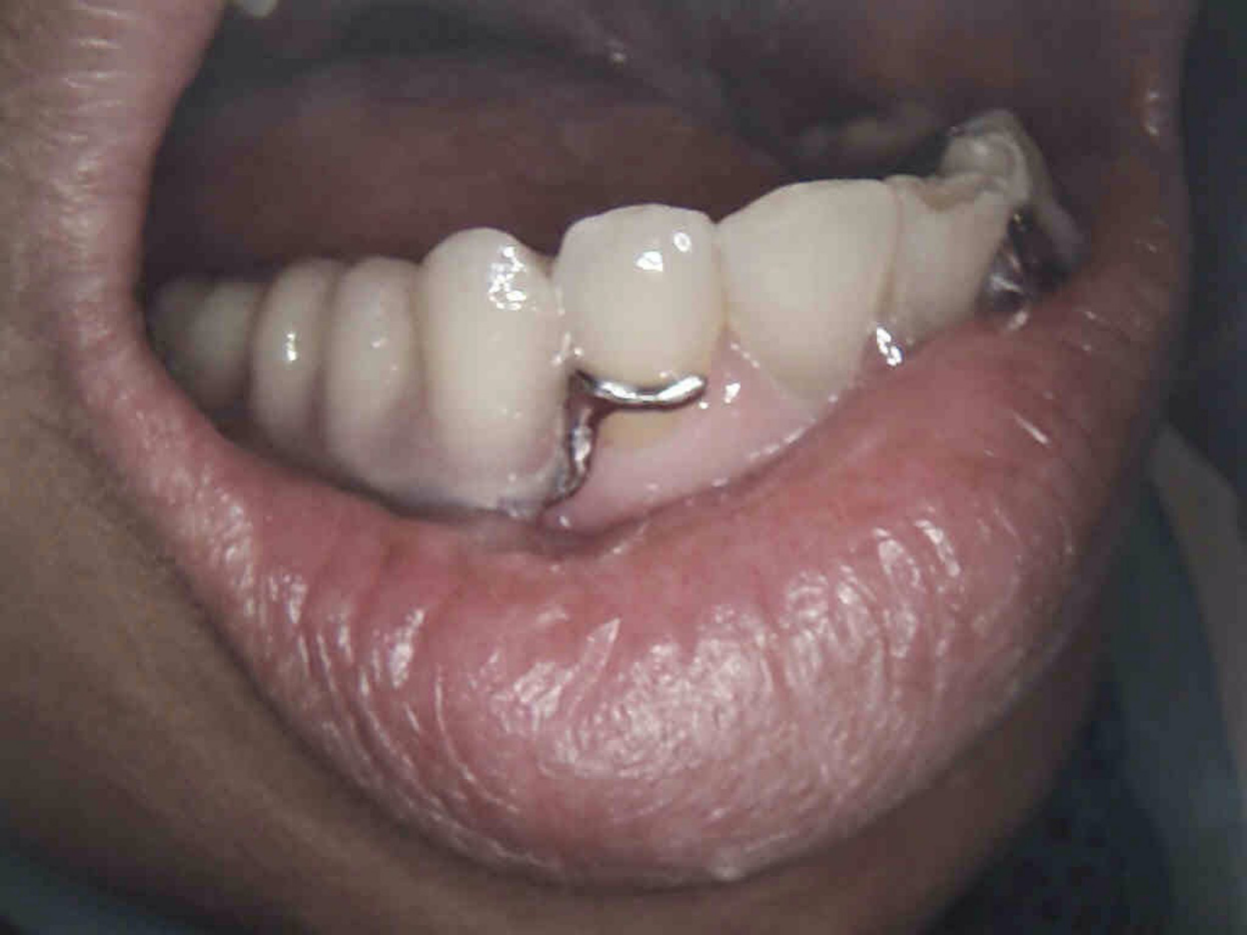
Definitive prosthesis inside the mouth.

The patient was followed-up after 24 h and 7 days after the procedure and then at follow-up appointments scheduled every 4 weeks for 1 year (Fig.5). Post-insertion follow-up showed fast adaptation to the newly inserted appliances with no ulcer formation, redness or lacerations. After 1 year of monthly follow-up, the patient's weight started to increase regularly at a rate of 1.8 to 2.2 kg per month.

## Discussion

There are number of conditions that can prevent a patient from sufficiently opening their mouth. The consequent risks of dry mouth, periodontal diseases, caries and even malnutrition are serious to those patients and can affect their quality of life if not treated properly.

Here, we have presented a case of a mandibulectomy patient who, after a successful surgery but without oral rehabilitation, suffered from subsequent severe weight loss because of malnutrition. Quality of life parameters including nutritional status and swallowing should be measured at diagnosis and at regular posttreatment intervals 13.

The patient was treated at an early stage of squamous cell carcinoma of the mandible. Early conservative surgery of the affected mandible is safer for patients with squamous carcinoma than carcinomas involving other sites (e.g., floor of the mouth) 19. Partial mandibulectomy can result in mandibular defects that require surgical and prosthetic intervention 20. During the prosthetic rehabilitation, consideration should be given to the prosthetic appliance that is used to replace the missing teeth, so as to properly distribute the occlusal forces to all remaining natural teeth and stress bearing areas 16. A thorough consideration should also be given to the masticatory efficiency of the patient because discontinuation of the mandible may lead patients to eat only foods that do not require a substantial amount of chewing 21.

The World Health Organisation (WHO) defines quality of life as an “individual's perception of their position in life in the context of the culture and value systems in which they live and in relation to their goals, expectations, standards, and concerns”. The key issues of HRQL in maxillofacial cancer patients are numerous and include coping, dental status, disfigurement, emotional problems and fear of recurrence 13. The principal complaint of our patient was a fear of recurrence as a psychological reaction to body weight loss in the first year after surgery. Absence of communication with the patient regarding the importance of oral rehabilitation leads the patient to develop high emotional anxiety that affected her daily performance.

## Conclusion

Our findings demonstrate that proper planning for oral rehabilitation should start immediately after completing mandibulectomy, and that this should be aimed at overcoming all unwanted consequences that may affect the patient's oral cavity and general health.
